# Reports on the Hospitals of the United Kingdom

**Published:** 1909-11-20

**Authors:** Henry Burdett


### REPORTS

ON THE

### Hospitals of the United Kingdom.

By SIR HENRY BURDETT, K.C.B., K.C.V.O.

### X.—SUSSEX COUNTY HOSPITAL, BRIGHTON.

This hospital was established in 1818. It contains 195 beds, of which upwards of 107 are occupied on an average. It has commanded the confidence and admiration of many wealthy visitors to Brighton, a circumstance which makes the large revenue it has received for many years from legacies one of the most remarkable features in the financial history of voluntary hospitals in this country.

So far as its buildings are concerned, this hospital represents no particular plan of construction, but has grown up and been reconstructed more times probably than any one now connected with it could accurately state. The site is airy and sunny, and good use has been made of it, so that the buildings have advantages which few town hospitals possess. The whole institution gives the visitor the impression of being one in which the Committee of Management take a genuine interest. A close inspection of the whole institution shows that there are still a few matters which earnestly demand immediate attention. The hygienic condition of the hospital, owing to the unusually large superficial space per bed, and to the absence of overcrowding, is good so far as the wards are concerned. There is, however, an absence almost everywhere of up-to-date lavatories, fittings, and baths. It is remarkable to notice the condition of the baths at this institution, where apparently cheapness rather than efficiency has been the guiding principle of those responsible for the existing baths, compared with that of the Rufford baths supplied probably thirty or thirty-five years ago to the Brighton Children's Hospital. At the Sussex County Hospital a number of the existing baths are quite unsuitable for hospital purposes, for they will not stand the wear and tear, and cannot be kept clean and attractive in appearance. All hospital baths should be porcelain, that is why Rufford's baths have such a deservedly high reputation for wear and appearance. Regarded as a modern hospital, the lavatories and ward kitchens are altogether out of date, but the best has been made of the accommodation, such as it is, and it is found sufficient.

Speaking generally, the ward floors are well kept, and the beds and bedding reasonably good, with the exception of flock pillows, which ought to find no place in a modern hospital. We observed, too, a want of order and system in the mattress department, which we believe would not continue if pensions held, as they ought to do, an adequate place in the scheme of administration adopted by the committee. We noticed, too, that there is great need for a linen department, which should be housed appropriately, so that abundance of space may be available for the handling, sorting, marking, mending, reception, and issue of all the linen and materials the property of the institution. We shall have something more to say on this head when we come to the laundry, but the committee must be quite aware that the existing needle-room is not a linen department, and the sooner they take steps to organise and equip one the better it will be for economic and administrative reasons.

### Housekeeping Department and Basement.

It is not accurate to speak of a basement at all in connection with these buildings. The basement so-called is really the ground floor of the buildings, and has been made to appear as a basement owing to the offices and entrance being placed on the first floor, in order, we presume, to make the façade as imposing as possible. Most of the rooms in this ground-floor basement are reasonably good, those at the back of the building, owing to the aspect, being colder and less attractive than the others. Here the wardmaids and domestic staff have their sleeping accommodation, and as far as we were able to judge there is not much to complain of, except perhaps that there is rather too little superficial space allotted in some of the rooms. The housekeeper has her office and departments here. She is a lady who was trained at Westminster, and displayed a most admirable knowledge of stores and their value, which ought to prove of great help in securing economy. We gather, however, that the housekeeper has nothing to do with the contracts, or the quality of the actual goods selected to be supplied by contract, which we think is a mistake. It is always well to take advantage of technical knowledge, and the present housekeeper would, we are confident, be able to give the Contract Committee many valuable hints if she were to attend their meetings when the contracts are given out, and to be invited to report upon the samples submitted. We are led to make these remarks from an inspection of the stores, and especially of the tea, which costs 1s. 3d. per lb., and is not in our judgment of sufficiently good quality for the money. We have seen much better teas in other hospitals which have been supplied at the same price, and we commend this fact to those whom it may concern. We noticed that the tins in which the patients' dinners are sent up to the wards are out of date and imperfect. Those we came across in the wards were quite cold, and we would suggest that steps should be taken to obtain the pattern of the tins in use at the Leicester Infirmary or the Nottingham General Hospital, where they have an excellent system, and every patient is supplied with hot meals, a point to which too much attention cannot be devoted where sick people are concerned.

### The Laundry Department.

The laundry is situated well away from the hospital, but it is not satisfactory. No adequate space is allotted for the reception of dirty linen, which is apt to accumulate near the entrance and presents an appearance certainly not attractive. No adequate provision, so far as we could find, exists either for the preliminary disinfection of foul linen, nor do the present arrangements of the laundry permit of its isolation and handling apart from the rest of the wash. The same remark applies to the sorting and despatch of the clean linen. There are no adequate shelves and racks, compartments for each ward, or baskets for each nurse and each member of the staff. Indeed, the laundry as it exists to-day must be troublesome and unnecessarily expensive to work, and its reconstruction should be promptly taken in hand. We spent some time in considering whether the existing buildings and the site on which they stand lend themselves to reconstruction at relatively small expense, so as to remove the existing objections and make them adequate for the efficient working of a laundry on a modern plan. We would venture to recommend that the drying-room behind the partition at the entrance to the laundry should be taken away, and that all the space thus secured should be thrown into the entrance lobby which should then, with the addition of shelves and baskets, be made adequate for the reception of the dirty linen and its proper distribution. Space could be found for the new drying-room in connection with the existing buildings by the exercise of a little ingenuity, and the room at the end of the ironing-room, which does not appear to fulfil any useful purpose, might be made available, if the dividing wall were taken down, for the purpose of sorting the clean linen and arranging it in its racks and baskets so as to prevent confusion, and expedite its distribution to the wards, nursing home, etc. The best idea of what a modern up-to-date laundry is, and how it should be organised and arranged can be gathered by an inspection of the new laundry buildings at the Coventry and Warwickshire Hospital, Coventry.

### Out-patient Department.

This department is situated in a separate building on a site opposite that upon which the main hospital buildings stand. The waiting hall is extensive, and every care appears to be exercised to facilitate the circulation of the patients and to see that the charity is not abused, as the almoner appears to go very thoroughly into her work, and to do her best to prevent any but those who really deserve hospital treatment from receiving it. We looked into some of the reports of the almoner and came to the conclusion, however, that a small committee might be appointed with advantage to supervise her reports, and to see that, whereas undeserving applicants must be excluded, all deserving and needy ones will be dealt with by being helped to obtain the relief and counsel which they often need. We will take one case which struck us forcibly. A domestic servant, a young woman apparently, weakened by ill-health, needed assistance to enable her to continue out of service, and, possibly requiring convalescent aid until her health was restored, was referred to the Charity Organisation Society. She does not appear to have availed herself of this advice, for the reason, we gathered, that she was feeling very unwell and could not face the ordeal. We further noticed that the hospital is on the telephone, and so is the C.O.S. It would seem a very simple matter for a poor woman of this kind to be helped by the hospital with the C.O.S., and for arrangements to be made which would save her much output of energy and difficulty by the use of the telephone and a little kindly co-operation. We may have hit upon one solitary instance in drawing this case out of a number which we found in the almoner's office; but our experience would lead us to think that this is not probable, and that there exists a field in this department for much good work on the part of one or two zealous members of the Committee, who will give a little personal service in a good cause.

The actual buildings and fittings of the out-patient department are well up to date, and many of them are admirable. It did not appear to us that the architect had so planned the adjuncts to the out-patient hall as to make the circulation of the patients as easy and convenient as is desirable. There was evidence, however, that the administration of the department by the staff was so planned as to overcome the difficulties created by the plan as far as practicable.

### Casualty Department.

This department is situated in a portion of the older buildings apart from the out-patient department, and leaves much to desire. Here adenoids and other cases are operated upon under conditions which, in our judgment, should be changed. There is no small operating theatre, fully equipped, as there ought to be, or recovery rooms, and we think if the Committee or some of its members were to attend in the casualty department and remain there on a busy day until all the operations were completed there is little doubt that a new casualty department would be forthcoming within a month.

### The Nursing Department.

We should say that from the hospital point of view the nursing department is one of the best administered and most perfect of all. From the nurses' point of view we are inclined to think that it offers certain injustices which should be abolished before a remedy is forthcoming under the law of supply and demand. Everywhere we found the sisters and nurses alert, intelligent, capable, zealous, and efficient. They keep their wards in perfect order; they look after their patients; and they show an admirable spirit of efficiency and *esprit* which must influence the work of the whole establishment. At this hospital it has been the practice for years not only not to pay the nurses, but to take a premium from the probationer when she first enters the service of the hospital. At the present time, owing to the law just referred to, no premiums are paid, nor are any salaries either. Having regard to the high quality of the certificates, and to the classes from which the nurses are drawn at the Sussex County Hospital, we feel this is a matter which ought to engage the immediate attention of the Nursing Committee, and that they should take steps to follow the example of other hospitals by paying their nurses a small salary during the first year, and making that salary increase up to the end of the third year, when certificates are issued. We fail altogether to see how a hospital can claim to be economically administered when it does not discharge the liability it undoubtedly has to the nursing staff, who ought no more to be asked to give their services free than ought any other members of the working staff.

There is a private nursing staff attached to the hospital, which is a good thing, but there are only 30 private nurses, and for financial reasons it is desirable that this staff shall be sufficient to meet the demand of the subscribers who need the services of a private staff. We hope, therefore, that steps may be taken to increase the number of private nurses engaged from 30 to 60. This would be good business for the reason that the trend of legislation in the present day is in the direction of destroying sympathy, and that must have an effect upon all institutions which depend upon voluntary aid for their support. Hospitals can, however, largely anticipate the effects of this danger by keeping a private nursing staff of sufficient dimensions to enable them to guarantee to every subscriber of a certain amount a private nurse when they may need one for themselves or members of their household. This arrangement must tend to increase largely the annual subscriptions of every hospital which adopts it, for the reason that an institution like the Sussex County Hospital, which trains its own nurses, can guarantee the character of every nurse attached to its private staff. This guarantee is an inestimable boon to private households who need nursing aid, and should be made the most of for financial and public reasons by every well-administered hospital in the country.

There is a nurses' home, which is a long walk up a steep incline, and is altogether inadequate for the needs and requirements of the nursing staff. Many of them, we understand, are at present housed in the town, an arrangement which has many disadvantages and nothing to recommend it. It is perfectly clear that the Committee of the Sussex County Hospital would be well advised to prepare a scheme for the erection of a sufficiently large and carefully planned nursing home, so that they may be in a position to house properly and care for the existing staff. Provision should also be made for the increase in the private nursing staff to which we have just referred. It struck us, too, that the nurses, especially as they have no pay whatever, are clearly entitled to greater consideration by the provision of more means of relaxation than they at present possess. There is only one tennis court, which, as far as we could ascertain, is seldom available for the nurses' use. The erection of an adequate nursing home would be a good opportunity to provide a scheme for indoor relaxation for nurses, and we hope this may be done with the aid of Captain Butler, the present chairman of the Nursing Committee.

### Finance.

The financial history of this hospital is remarkable. The accounts for the year 1908 put the position in a nutshell. The total ordinary expenditure amounted to £16,279, whilst the ordinary income, excluding income from investments (£3,000), was under £6,000, or rather more than one-third of the ordinary expenditure. The net deficiency between ordinary income and ordinary expenditure was upwards of £7,000, but the accounts exhibit an excess of total income over total expenditure for the year of upwards of £8,000. The explanation is that the Sussex County Hospital has had for very many years a splendid revenue from legacies. These amounted to £15,642 in 1908. Taking the ten years ending 1908, legacies have yielded an average revenue of nearly £10,000 a year. This unique experience cannot be expected to continue, and wisdom should spur the management on to appoint an active Finance Committee to look into the financial condition of this hospital, which cannot, from the point of view of a business man, be regarded as satisfactory. The hospital has upwards of £100,000 invested, but the yield from annual subscriptions is remarkably small, having regard to the circumstances of the inhabitants of the district which this institution serves. We note, too, that the annual subscriptions are steadily falling off, the revenue from this source having amounted to £2,645 in 1898, against £2,423 in 1908. The history of voluntary hospitals proves that these figures may be taken as evidence that there is an absence of financial grip and direction, due probably to the feeling that legacies will keep the hospital going, and that it is therefore not necessary to worry about the financial position. If any such feeling exists, it constitutes a very real danger to the permanent maintenance of this great charity, and the sooner it is ended or mended the better. There is no reason whatever why the income from voluntary sources of this hospital should not be raised to £10,000 a year, if the experience of other similar institutions may be accepted as an indication. We do not propose to-day to go into this point in detail, but it is one which calls for the immediate attention of all well-wishers of the Sussex County Hospital, for sound finance is the foundation of material prosperity, and no hospital can endure, or be efficiently administered, without it.

---

**The South Devon and East Cornwall Hospital Plymouth,** will be reported on next week.

